# Frequent Undetected Ward-Based Methicillin-Resistant *Staphylococcus aureus* Transmission Linked to Patient Sharing Between Hospitals

**DOI:** 10.1093/cid/cix901

**Published:** 2017-10-31

**Authors:** Olga Tosas Auguet, Richard A Stabler, Jason Betley, Mark D Preston, Mandeep Dhaliwal, Michael Gaunt, Avgousta Ioannou, Nergish Desai, Tacim Karadag, Rahul Batra, Jonathan A Otter, Helene Marbach, Taane G Clark, Jonathan D Edgeworth

**Affiliations:** 1 Centre for Clinical Infection and Diagnostics Research, Department of Infectious Diseases, King’s College London and Guy’s and St Thomas’ NHS Foundation Trust; 2 Oxford Health Systems Collaboration, Centre for Tropical Medicine and Global Health, Nuffield Department of Clinical Medicine, University of Oxford; 3 Department of Pathogen Molecular Biology, Faculty of Infectious and Tropical Diseases, London School of Hygiene and Tropical Medicine; 4 Illumina, Cambridge Ltd, Chesterford Research Park, Little Chesterford, Essex; 5 Department of Medical Microbiology, King’s College Hospital NHS Foundation Trust; 6 Department of Microbiology, University Hospital Lewisham, Lewisham and Greenwich NHS Trust; 7 National Institute for Health Research Health Protection Research Unit in Healthcare-Associated Infections and Antimicrobial Resistance at Imperial College London, and Imperial College Healthcare NHS Trust, Infection Prevention and Control; 8 Faculty of Epidemiology and Population Health, London School of Hygiene and Tropical Medicine, London, United Kingdom

**Keywords:** MRSA, transmission, whole-genome sequencing, healthcare network, patient sharing

## Abstract

**Background:**

Recent evidence suggests that hospital transmission of methicillin-resistant *Staphylococcus aureus* (MRSA) is uncommon in UK centers that have implemented sustained infection control programs. We investigated whether a healthcare-network analysis could shed light on transmission paths currently sustaining MRSA levels in UK hospitals.

**Methods:**

A cross-sectional observational study was performed in 2 National Health Service hospital groups and a general district hospital in Southeast London. All MRSA patients identified at inpatient, outpatient, and community settings between 1 November 2011 and 29 February 2012 were included. We identified genetically defined MRSA transmission clusters in individual hospitals and across the healthcare network, and examined genetic differentiation of sequence type (ST) 22 MRSA isolates within and between hospitals and inpatient or outpatient and community settings, as informed by average and median pairwise single-nucleotide polymorphisms (SNPs) and SNP-based proportions of nearly identical isolates.

**Results:**

Two hundred forty-eight of 610 (40.7%) MRSA patients were linked in 90 transmission clusters, of which 27 spanned multiple hospitals. Analysis of a large 32 patient ST22-MRSA cluster showed that 26 of 32 patients (81.3%) had multiple contacts with one another during ward stays at any hospital. No residential, outpatient, or significant community healthcare contacts were identified. Genetic differentiation between ST22 MRSA inpatient isolates from different hospitals was less than between inpatient isolates from the same hospitals (*P* ≤ .01).

**Conclusions:**

There is evidence of frequent ward-based transmission of MRSA brought about by frequent patient admissions to multiple hospitals. Limiting in-ward transmission requires sharing of MRSA status data between hospitals.

## Teaser

We report significant undetected ward-based transmission of methicillin-resistant *Staphylococcus aureus* in the English national health system, which is predominantly attributable to patient sharing between hospitals. We propose a context-appropriate measure that could limit the spread of antibiotic-resistant bacteria in England.

Infection prevention and control efforts have resulted in many countries reporting large reductions in endemic methicillin- resistant *Staphylococcus aureus* (MRSA) [1, 2]. In England, this reduction has been achieved through a single-hospital-centric approach to epidemiology, prevention, and control [2].

Recent evidence suggests limited within-hospital transmission of *S. aureus* based on genetic relatedness of inpatient isolates [3–5]. However, there is also evidence that frequent patient admissions to multiple hospitals in a network results in regional spread of antibiotic-resistant bacteria, including MRSA [6–13]. In one US study, MRSA genetic relatedness in patients at different hospitals correlated with the percentage of patient sharing between those hospitals [9]. This suggests there may be benefit in moving from a single-hospital to a healthcare-network approach to surveillance and implementation of infection prevention and control measures, to gain further reductions in MRSA [14, 15]. However, further information is required to inform the establishment of healthcare-network interventions.

We analyzed a collection of MRSA isolates and linked metadata from patients attending a network of linked hospitals and surrounding primary healthcare facilities in Southeast London at a time when mandatory universal MRSA admission screening was in place. All hospitals had reported sustained stable control of MRSA over at least the preceding 5 years, in line with national trends [2]. The objective was to identify whether and where MRSA transmission was occurring by taking a healthcare-network rather than single-hospital approach to surveillance.

## METHODS

### Study Population

**Figure 1.**
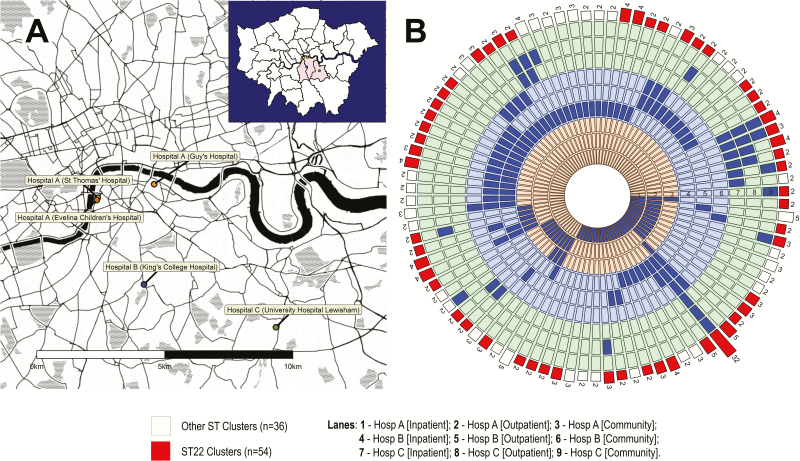
Map of hospitals and distribution of 90 genetically defined transmission clusters across services in the hospital cohort. *A*, Upper right corner shows a map of London, with the catchment area boroughs served by participant hospitals shaded in pink (Southwark, Lambeth, and Lewisham). Hospital A (Guy’s and St Thomas’ National Health Service [NHS] Foundation Trust) comprises 3 hospitals, just south of the river Thames. Hospital B corresponds to King’s College NHS Foundation Trust, and comprises 1 hospital. Hospital C comprises University Hospital Lewisham, which is now part of the Lewisham and Greenwich NHS Trust. *B*, The 9 concentric circles represent healthcare settings (ie, inpatient; outpatient; community) for hospitals A (orange), B (blue), and C (green). The 90 transmission clusters are represented by a segment. The number of unique patients in the cluster is shown by a bar. Red and white bars correspond to transmission clusters of sequence type (ST) 22 and other STs, respectively. For each cluster, the location of patients in the cluster at the time of sampling is shown in blue. For example, 32 patients in the largest cluster where identified across 7 of 9 hospital settings. Twenty-nine of 90 transmission clusters (32.2%) occurred within a single hospital setting. Sixty-one clusters (67.8%) involved >1 setting in either a single hospital (n = 34 [37.8%]; median = 2 settings) or in multiple hospitals (n = 27 [30.0%]; median = 2 hospitals). Abbreviation: ST, sequence type.

A description of the study population has been reported previously [16, 17]. All MRSA isolates identified over 4 months (November 2011 to February 2012) were collected from 3 National Health Service (NHS) hospital microbiology laboratories collectively serving a population of 867254 usual residents [18] and providing service to all inpatients, outpatients, and surrounding primary community-based healthcare facilities (ie, general practices [GPs]) in Lambeth, Southwark, and Lewisham boroughs of Southeast London. Participating centers comprised 4 acute tertiary-referral hospitals in 2 NHS hospital groups (Guy’s and St Thomas’ NHS Foundation Trust [GSTT; hospital A] and King’s College NHS Foundation Trust [hospital B]) plus 1 acute District General Hospital (University Hospital Lewisham [hospital C]). Hospital A (GSTT) comprises St Thomas’ Hospital, Guy’s Hospital, and the London Evelina Children’s Hospital (Figure 1A). Patient transfers and referrals between participant hospitals are common, and the hospitals’ overlapping catchment areas mean that local usual residents may be admitted to any hospital without referral. A system for sharing clinical patient details between healthcare facilities was not available at the time of the study, implying that history of MRSA from other facilities was not obviously available for patients admitted without referral. All acute tertiary-referral sites regularly provide services to international patients and those from other London boroughs and sites in the United Kingdom, which are not discharged back into the catchment areas of the hospital cohort. All participant hospitals had in place MRSA universal admission screening plus weekly follow-up screening for inpatients in high-risk units at the time of the study.

### Laboratory Methods

Isolates from participant hospitals were submitted to the Centre for Clinical Infection and Diagnostics Research at GSTT for an independent identity check and included in the study if confirmed as MRSA by culture on chromogenic agar (Oxoid Brilliance) and rapid latex agglutination test (Staphaurex, Remel) [16, 17]. Relevant patient-level metadata submitted with each specimen included the patient’s residential postcode, the identifier and postcode of the general practice routinely providing primary care to the patient, and the postcode and unique identifier of the clinical location managing the patient at the time of sampling. Clinical location data were aggregated for analysis into settings (defined as inpatient, outpatient, or community) linked to each participant hospital. Using GeoConvert [19], postcode data were converted to lower super output areas (LSOAs) for analysis. LSOAs are boundary data for small geographies, which comprise 1500 residents and 650 households on average [18].

Whole-genome sequencing (WGS) was conducted at Illumina UK on the first confirmed MRSA isolate from each patient at each unique clinical location over the study period as described previously [16, 17].

### Analysis of WGS Data

Sequence data were mapped to the EMRSA-15 reference genome MRSA252 (GenBank accession number BX571856, ST36) using BWA mem [20] in agreement with standard practice [21]. Application of SAMtools software suite [22] identified 152 797 high-quality single-nucleotide polymorphism (SNP) positions (quality score ≥30; 1 error/1000 base pairs). SNP genotypes (alleles) were called based on a minimum 10-fold allelic coverage or considered missing [23]. SNPs in non-unique genomic regions were removed. Additional filtering based on missing genotypes was applied to both SNPs and samples, leading to a final analysis dataset and 68997 (45.2%) high-quality SNP loci per sample. The proportion of missing genotypes across the dataset was low (0.1%). The SNPs covered the core MRSA genome [24] (≥1 SNP in 2099 of 2744 genes), with the majority located in genic regions (87.7%). Thirty-nine percent of mutations were nonsynonymous. To describe local MRSA phylogenies and clade informative variation, SNP data were used to create best-scoring maximum likelihood phylogenic trees using RAxML, with default settings and 1000 bootstrap samples [25].

To determine the sequence-typing classification of each isolate, draft genome assemblies were compared to custom BLAST databases for each of the pubMLST multilocus sequence typing (MLST) alleles. Allele BLAST outputs were filtered to identify sequences with 100% identity across the entire allele length, and the sequence type (ST) was determined using the combination of alleles. To identify staphylococcal cassette chromosome *mec* (SCC*mec*), reference BLAST databases of *ccr* and *mec* gene complexes were constructed from known sccMEC types (accession numbers AB033763, AB037671, AB063172, AB063173, AB096217, AB097677, AB121219, AB373032, AB425823, AB425824, AB505628, AB505630, AF411935, D86934, DQ106887, FJ670542). Draft assemblies were compared to the BLAST databases and outputs were filtered to determine the *ccr* and *mec* complexes. Unusual *ccr* and *mec* complexes or absent/poor data were confirmed by mapping raw sequence data against SCC*mec* references and visually inspecting each alignment. SCC*mec* types were determined using International Working Group on the Staphylococcal Cassette Chromosome elements definitions (http://www.sccmec.org/Pages/SCC_TypesEN.html).

### Genetically Defined Clusters

Clusters were identified based on pairwise SNP differences between isolates without prior knowledge of patient location or epidemiological linkages. The SNP cutoff to define clusters (≤10 SNP) was based on the observed multimodal distributions of inter- and intraperson SNP differences (Supplementary Figure 1 and Supplementary Table 1) and the numbers of clusters and cluster sizes identified when considering 0, ≤5, 10, 15, or 20 SNP cutoffs, which showed the number of clusters peaking at ≤10 SNP (Supplementary Table 2). The frequency of patient-to-patient transmission events within the study population was inferred by counting the number of unique patients in each possible transmission cluster (eg, 2 transmission events inferred from a cluster of 3 patients). The total number of patients linked to transmission was calculated by de-duplicating patients represented in >1 cluster. We compared numbers and sizes of clusters and numbers of patients linked to transmission when restricting the analysis to individual hospitals in isolation compared with considering all healthcare settings combined.

### Epidemiological Linkage of Patients in Clusters

When considering all healthcare settings combined, we examined whether patients in the largest cluster could be linked by their residential LSOA or by routinely attending the same community-based general practice, having attended the same outpatient clinic at the same time during the study period or the preceding 12 months, or having stayed in the same inpatient ward across any of the 3 hospitals at the same time or within 7 days of each other during the study period or the preceding 12 months. We allowed this 7-day gap to account for indirect transmission via a staff carrier or contaminated surface [3].

### Genetic Differentiation of MRSA

We calculated the proportion of nearly identical ST22 isolates (*I*) from patients living within the catchment area [7] to compare genetic differentiation of MRSA within and between hospitals and community settings [7]. For robustness, “*I*” was determined by the proportion of isolate pairs with ≤10, 20, 30, or 40 SNPs because various thresholds have been used to discount direct transmission [3, 24]. We further validated the transmission dynamics inferred from “*I*,” by comparing average and median pairwise SNP differences (π) across isolates within and between hospital inpatient settings and surrounding communities [7]. The statistical significance of genetic differentiation of MRSA populations was assessed through permutation tests over 10000 random permutations of clinical location identifiers relative to the list of isolates [7].

WGS data are available from the European Nucleotide Archive database under accession number PRJEB11177 [17]. Patient-level metadata are available from the corresponding author.

### Ethical Considerations

This research was conducted following approval from the National Research Ethics Service (reference: 11/NW/0733). Approval and waived consent was obtained from NHS research and development departments at participating hospitals.

## RESULTS

### Study Demographics

A total of 68997 high-quality genome-wide SNPs were analyzed for 685 isolates from 610 patients, of which 56.1% (n = 342) were usual residents of the cohort boroughs (Figure 1A). Review of each hospital’s own records identified 48.2% (n = 293) of patients having been admitted to that hospital during the 12 months preceding their enrollment in the study, 38.2% (n = 233) having previously been identified with MRSA, and 20.3% (n = 124) having their first MRSA identification within the year preceding their enrollment (Table 1).

**Table 1.** Characteristics of Patients With Methicillin-Resistant *Staphylococcus aureus*

Characteristic	Hospital A (n = 256)	Hospital B (n = 251)	Hospital C (n = 103)	Total (N = 610)
Age, y, mean (SD)	54.4 (25.5)	58.5 (23.1)	69.3 (19.4)	58.6 (24.1)
Age group				
≤14 y	9.4 (24)	5.2 (13)	1.0 (1)	6.2 (38)
≥65 y	41.4 (106)	43.8 (110)	66.0 (68)	46.6 (284)
Sex, female	41.0 (105)	37.5 (94)	51.5 (53)	41.3 (252)
Residential LSOA within catchment boroughs	48.4 (124)	55.0 (138)	77.7 (80)	56.1 (342)
>1 healthcare episode during the study	24.2 (62)	23.5 (59)	17.5 (18)	22.8 (139)
Isolates with sequence data per patient^a^				
1	89.5 (229)	87.6 (220)	90.3 (93)	88.9 (542)
2	8.6 (22)	11.6 (29)	9.7 (10)	10.0 (61)
3	2.0 (5)	0.8 (2)	0.0 (0)	1.1 (7)
Type of healthcare episode				
Inpatient only	54.3 (139)	51 (128)	19.4 (20)	47.0 (287)
Outpatient only	21.5 (55)	27.5 (69)	40.8 (42)	27.2 (166)
Community only	7.8 (20)	8.8 (22)	27.2 (28)	11.5 (70)
Mixed	16.4 (42)	12.7 (32)	12.6 (13)	14.3 (87)
Had previous history of MRSA	32.8 (84)	43.4 (109)	38.8 (40)	38.2 (233)
Time to first MRSA diagnosis				
≤1 y	18.4 (47)	24.3 (61)	15.5 (16)	20.3 (124)
>1–5 y	8.6 (22)	12.4 (31)	16.5 (17)	11.5 (70)
>5 y	5.9 (15)	6.8 (17)	6.8 (7)	6.4 (39)
Had previous admission to hospital	63.7 (163)	65.3 (164)	68.3 (69)^b^	65.1 (396)^b^
Time to previous admission				
≤1 y	52.3 (134)	47.8 (120)	38.6 (39)^b^	48.2 (293)^b^
>1–5 y	5.9 (15)	12.4 (31)	16.8 (17)^b^	10.4 (63)^b^
>5 y	5.5 (14)	5.2 (13)	12.9 (13)^b^	6.6 (40)^b^

Data are presented as percentage (No.) unless otherwise indicated.

Abbreviations: LSOA, lower super output area; MRSA, methicillin-resistant *Staphylococcus aureus*; SD, standard deviation.

^a^Fifty-four patients had isolates sequenced in multiple settings (43 [inpatient and outpatient]; 5 [inpatient and community]; 5 [outpatient and community]; 1 [inpatient, outpatient, and community]) and 20 had (instead or in addition) >1 isolate (maximum 2) sequenced within the same hospital setting.

^b^Information on previous admission to hospital was missing for 2 patients in hospital C. Hospital C and total denominators were therefore 101 and 608, respectively.

Most patients (542 [88.9%]) contributed a single sequenced isolate, but 11.1% (n = 68) contributed more (maximum of 3). Five hundred twenty-three (85.7%) patients had MRSA-positive healthcare episodes in a single setting (inpatient: 47.0%; outpatient: 27.2%; community: 11.5%). Most isolates (352 [51.4%]) were from inpatients (Table 2).

**Table 2.** Numbers of Isolates, Genetically Defined Clusters, Inferred Transmission Events, and Patients Linked to Transmission Events by Methicillin-Resistant *Staphylococcus aureus* Multilocus Sequence Type

	Total	ST22	ST36	ST8	ST5	ST1	ST88	Other ST
Isolates								
Total	100 (685)	59.6 (408)	9.9 (68)	5.5 (38)	3.6 (25)	3.5 (24)	2.3 (16)	15.5 (106)
Inpatient settings	51.4 (352)	52.7 (215)	67.6 (46)	52.6 (20)	32.0 (8)	37.5 (9)	50 (8)	43.4 (46)
Hospital A	48.6 (171)	57.9 (99)	9.9 (17)	10.5 (18)	2.9 (5)	4.1 (7)	0.6 (1)	14.0 (24)
Hospital B	42.9 (151)	61.6 (93)	17.9 (27)	1.3 (2)	2.0 (3)	1.3 (2)	4.6 (7)	11.3 (17)
Hospital C	8.5 (30)	76.7 (23)	6.7 (2)					
Outpatient settings	35.5 (243)	34.6 (141)	26.5 (18)	34.2 (13)	52.0 (13)	37.5 (9)	37.5 (6)	40.6 (43)
Hospital A	35.8 (87)	46.0 (40)	4.6 (4)	9.2 (8)	6.9 (6)	5.7 (5)	3.4 (3)	24.1 (21)
Hospital B	42.8 (104)	56.7 (59)	12.5 (13)	3.8 (4)	3.8 (4)	3.8 (4)	2.9 (3)	16.3 (17)
Hospital C	21.4 (52)	80.8 (42)	1.9 (1)	1.9 (1)	5.8 (3)			9.6 (5)
Community settings	13.1 (90)	12.7 (52)	5.9 (4)	13.2 (5)	16.0 (4)	25.0 (6)	12.5 (2)	16 (17)
Hospital A	33.3 (30)	56.7 (17)	10.0 (3)	6.7 (2)		6.7 (2)	3.3 (1)	16.7 (5)
Hospital B	32.2 (29)	48.3 (14)	3.4 (1)	6.9 (2)	6.9 (2)	6.9 (2)		27.6 (8)
Hospital C	34.4 (31)	67.7 (21)		3.2 (1)	6.5 (2)	6.5 (2)	3.2 (1)	12.9 (4)
Clusters (≤10 SNPs)								
Isolate clusters (> 1 isolate)	100 (115)	60.0 (69)	13.0 (15)	4.3 (5)	3.5 (4)	3.5 (4)	3.5 (4)	12.2 (14)
Patient clusters (> 1 patient)	78.3 (90)	60.0 (54)	14.4 (13)	4.4 (4)	4.4 (4)	2.2 (2)	3.3 (3)	11.1 (10)
Isolates in patient clusters	41.9 (287)	64.8 (186)	12.9 (37)	4.2 (12)	3.8 (11)	1.4 (4)	2.4 (7)	10.5 (30)
Size of transmission clusters								
2 patients	61.1 (55)	56.4 (31)	14.5 (8)	5.5 (3)	5.5 (3)	3.6 (2)	3.6 (2)	10.9 (6)
3 patients	23.3 (21)	57.1 (12)	14.3 (3)	4.8 (1)	4.8 (1)		4.8 (1)	14.3 (3)
4 patients	10.0 (9)	88.9 (8)	11.1 (1)					
5 patients	4.4 (4)	50.0 (2)	25.0 (1)					25 (1)
32 patients	1.1 (1)	100 (1)						
Transmission events^a^								
Within hospital settings (Σ)	102	67.6 (69)	15.7 (16)	2.9 (3)	2.0 (2)	1.0 (1)	3.9 (4)	6.9 (7)
Within and between hospital settings (Σ)	171	69.0 (118)	12.3 (21)	2.9 (5)	2.9 (5)	1.2 (2)	2.3 (4)	9.4 (16)
Patients linked to transmission events^b^								
Within hospital settings (Σ)	26.4 (161)	67.1 (108)	15.5 (25)	3.1 (5)	2.5 (4)	1.2 (2)	4.3 (7)	8.1 (13)
Within and between hospital settings (Σ)	40.7 (248)	66.9 (166)	13.7 (34)	3.6 (9)	3.6 (9)	1.6 (4)	2.8 (7)	10.5 (26)

Data are presented as percentage (No.) unless otherwise indicated.

Abbreviations: SNP, single-nucleotide polymorphisms; ST, sequence type.

^a^Patient-to-patient transmission events were inferred from counting the number of unique patients in each cluster (eg, 2 transmission events inferred from a cluster with 3 patients). The same cluster may have been composed of >3 isolates, if any of the patients had >1 isolate in the cluster.

^b^Numbers of patients linked to transmission events were calculated by de-duplicating patients represented in >1 cluster. Total percentages for isolates in transmission clusters and patients linked to transmission events are based on 685 isolates and 610 patients, respectively.

### MRSA MLST and Population Phylogeny

MLST identified 31 STs, with more than half of isolates being ST22 (n = 408 [59.6%]), followed by ST36 (n = 68 [9.9%]) and ST8 (n = 38 [5.5%]). Fifteen novel STs were identified from 22 isolates (3.2%), of which 14 of 15 deviated by single SNP variants from known STs (predominantly ST22s). A SNP phylogenetic tree clustered all STs into 3 clades (Supplementary Figure 2).

### Clusters in Single and Multiple Hospital Settings

Linking isolates based on ≤10 SNP difference identified 90 clusters involving ≥2 patients that formed possible transmission chains (Figure 1B); of these, 61.1% (n = 55) involved 2 patients, whereas the remaining clusters involved 3 (n = 21 [23.3%]), 4 (n = 9 [10.0%]), 5 (n = 4 [4.4%]), or 32 (n = 1 [1.1%]) patients (Table 2). The majority of transmission clusters involved individuals in inpatient wards (n = 70 [77.8%]), with most (n = 61 [67.8%]) spanning multiple settings within a hospital (n = 34 [37.8%]) or across >1 hospital (n = 27 [30.0%]) (Figure 1B).

**Figure 2.**
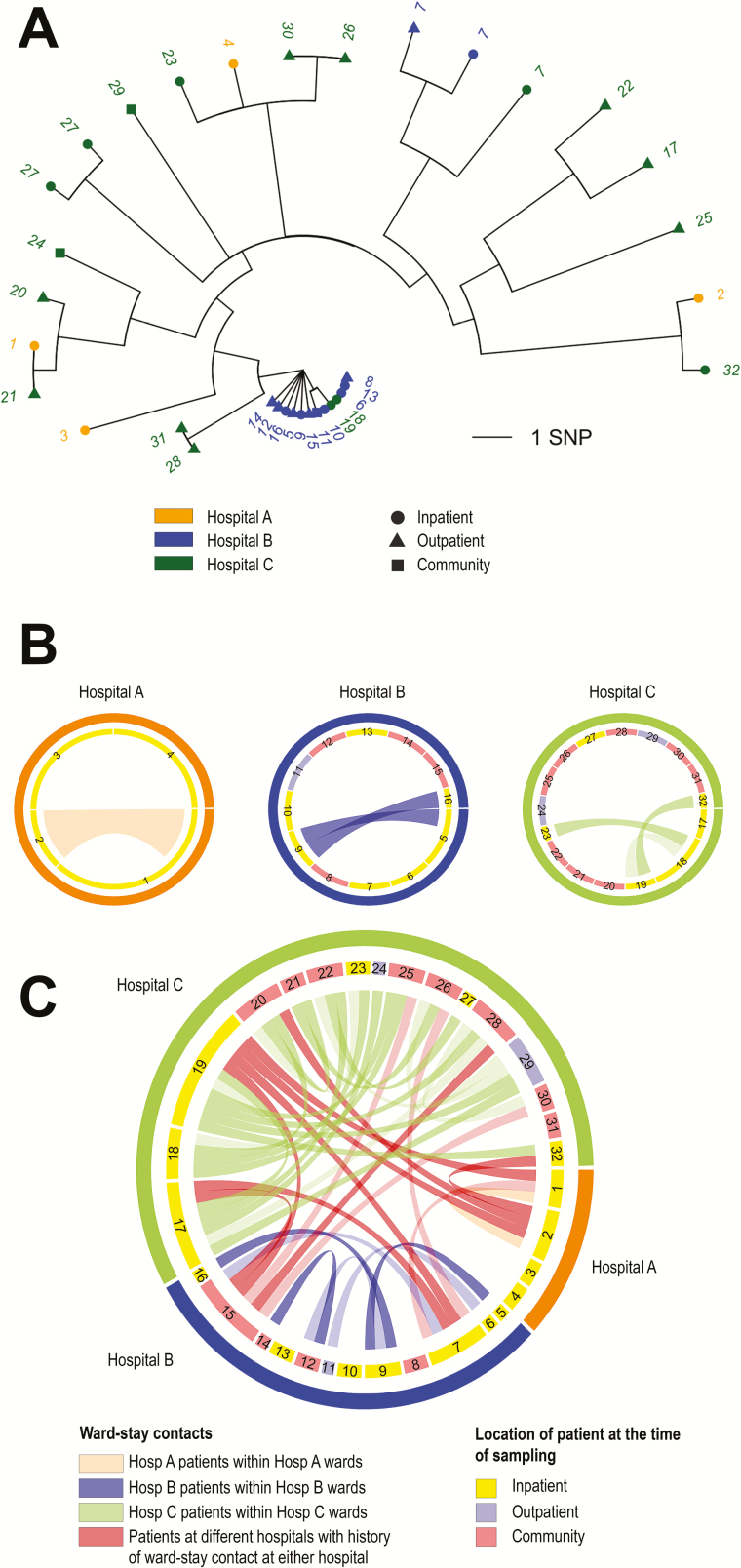
Transmission cluster of genetically related sequence type (ST) 22 methicillin-resistant *Staphylococcus aureus* (MRSA): combined analysis of single-nucleotide polymorphism (SNP) and epidemiological data from single-hospital inpatient settings compared with analysis of data across the healthcare network. *A*, SNP phylogeny for the larger ST22 transmission cluster, which comprised 35 isolates from 32 patients in hospitals A (n = 4), B (n = 12), and C (n = 16). Numbers refer to patient identifiers. The SNP phylogeny identified 6 identical isolates from hospitals B (n = 4) and C (n = 2), 7 isolates from hospital B within 1 SNP of each other and the central core, and an additional 22 isolates (predominantly from hospital C) no more than 10 SNPs different from each other and from the central core. These 22 isolates included 2 pairs of unique-patient isolates from hospitals A and C (patients 1 and 21; patients 30 and 26), which differed by 5 SNPs from the core. Same-patient isolates were taken from 1 individual in hospital B (patient 7), while moving between inpatient and outpatient settings. Two additional repeat isolates were from 1 inpatient in hospital C (patient 27). The phylogeny identifies transmission across settings in the cohort based on relatedness of isolates alone. For example, 2 identical isolates from hospital A (inpatient 1) and C (outpatient 21) were related to central core isolates by outpatient 20 in hospital C. Diagrams in *B* and *C* show inpatient-ward contacts between patients in the larger transmission cluster. *B*, A case where each hospital analysis combined SNP and ward-stay data from their own inpatient setting alone. *C*, Contacts identified when the analysis considers all settings and hospitals combined. Patients are represented by a segment and identified with the same number as that shown in the SNP phylogeny. Individuals who were admitted to the same ward during the study period or the preceding 12 months are linked to one another through a band, the transparency of which indicates the timing of the contact (ie, < transparent: in the same ward at the same time; > transparent: in the same ward within a week of each other). Bands that are the same color as the hospital segment indicate contacts between patients identified by that hospital that occurred in wards at that same hospital. Red bands show contacts between patients from different hospitals, who were found to have stayed in the same ward as a result of patient transfers or referrals. Admission of 4 individuals from hospital A (patient 1 and 2) and B (patient 7 and 15) to hospital C (bottom diagram) facilitated at least 46 in-ward contacts among the 32 patients across the 3 hospitals. Two of 6 patients without contacts (bottom diagram) were babies whose mothers’ history was not traced. Analyses limited to inpatients from each hospital separately identified half of MRSA acquisitions (17 inpatients of 32 patients [53.1%]), 13.0% of ward-stay contacts (6/46), and 19.4% (n = 6/31) of unique patient-to-patient transmission events in the cluster. Abbreviation: SNP, single-nucleotide polymorphism.

The largest cluster comprised 32 patients with ST22-MRSA identified at hospitals A (n = 4), B (n = 12), and C (n = 16), spanning 7 of the 9 settings in the cohort (Figures 1B and 2A).

Over the 4-month period, 40.7% of patients (248/610) could be linked to 1 of at least 171 possible transmission events (Table 2). Confining analysis to each hospital setting separately missed 40.4% (69/171) of possible transmission events and 35.1% (87/248) of patients linked to transmission across the hospital network (Table 2).

### Epidemiological Linkage Within and Between Hospital Settings of Patients in the Large ST22 Transmission Cluster

Data on inpatient and outpatient contact during the preceding year, residential postcode, and community healthcare provider were obtained from the 32-patient ST22 cluster to identity the likely setting of MRSA transmission. First, patient data were analyzed separately from each hospital, which epidemiologically linked only 9 patients (2, 3, and 4 inpatients from hospitals A, B, and C, respectively) based on criteria outlined earlier. This would indicate ≤3 inpatient transmission events at any hospital (total 6) and the likely conclusion that in-hospital transmission was uncommon (Figure 2B). However, combining the analysis across all settings in the network, a complex pattern of frequent hospital stay emerged, which showed that 26 of 32 patients (81.3%) had multiple contacts with one another during ward stays at any hospital (Figure 2C). No outpatient contacts were identified, no 2 individuals shared the same residential LSOA and no more than 2 shared the same GP registration, suggesting that transmission occurred on hospital wards.

#### Genetic Differentiation of ST22 MRSA Within and Between Hospital Inpatient Settings

To understand the impact of patient sharing between hospitals on in-hospital transmission, we analyzed the genetic differentiation of ST22 MRSA within and between hospital inpatient settings. We considered only isolates from patients with residential LSOA within the catchment boroughs (n = 230/408 ST22 isolates) to avoid bias arising from nonlocal patients accessing inpatient or outpatient care but not primary care in the area and being less likely to be admitted to >1 hospital locally.

**Figure 3.**
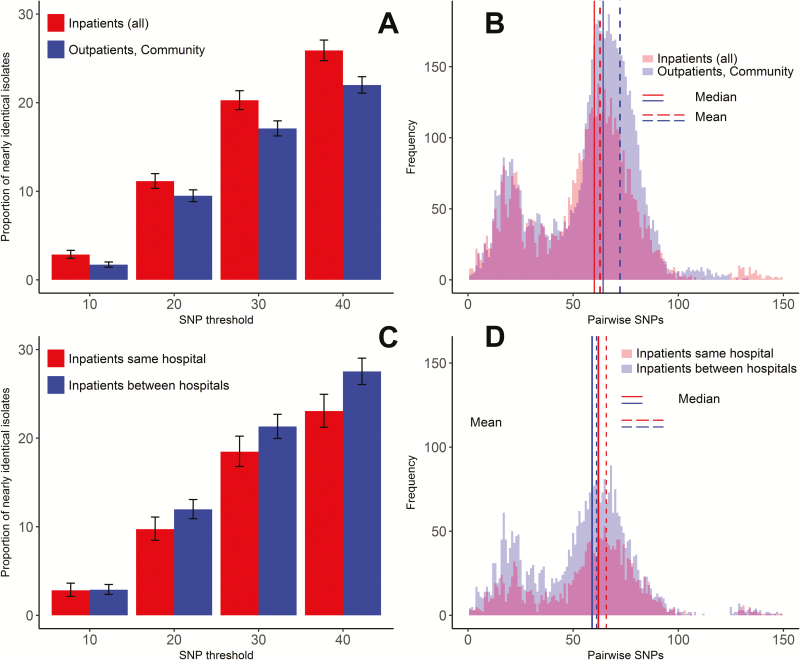
*A–D*, Nearly identical isolates and average pairwise single-nucleotide polymorphisms for sequence type 22 isolates from usual residents in inpatient settings within a same hospital or different hospitals and in community or outpatient settings. Abbreviation: SNP, single-nucleotide polymorphism.

Median and average pairwise SNP differences between isolates from inpatient settings across the cohort were significantly smaller than those from community and outpatient settings combined (mean: 63 vs 72, *P* = .0001; median = 60 vs 64, *P* = .0001) (Figure 3B). The proportions of nearly identical isolates were also consistently higher among inpatient isolates at ≤10, 20, 30, and 40 SNPs (2.9% vs 1.7%, *P* = .0001; 11.0% vs 9.5%, *P* = .0026; 20.3% vs 17.1%, *P* = .0001; 25.9% vs 22.0%, *P* = .0001) (Figure 3A).

The average and median pairwise genetic distance between inpatient isolates from the same hospitals was significantly larger than between isolates from different hospitals (mean: 66 vs 61, *P* = .0003; median = 62 vs 59, *P* = .0001) (Figure 3D). Proportions of nearly identical isolates at the ≤10 SNPs cutoff were similar (2.8% vs 2.9%, *P* = .8743), but those at ≤20, 30, and 40 SNPs were significantly higher for isolates from different hospitals (9.7% vs 12.0%, *P* = .011; 18.5% vs 21.3%, *P* = .0101; 23.1% vs 27.5%, *P* = .0002) (Figure 3C). The results of the analyses were consistent with those considering only the first isolate from each patient across the healthcare network.

## DISCUSSION

This study found compelling evidence for ward-based MRSA transmission linked to patient sharing across a network of hospitals, each of which had implemented a successful infection control program over many years and seen rates fall by >90% from their peak. Confining analysis to each hospital setting separately missed 40.4% (69/171) of possible transmission events across the hospital network. Analysis of inpatient data from each hospital separately would not have reached the same conclusion, because many of the linked patients were only identified by samples obtained when they contacted other hospitals or community healthcare facilities. This was highlighted through analysis of the 32-patient ST22 cluster. Separate analysis of each hospital’s inpatient-obtained samples would have identified only 9 of 32 (28%) patients linked to possible transmission events based on epidemiological and genetic observations. But when hospital-stay records from the 32 patients were considered together across all participating hospitals and settings, a complex network of ward contacts was revealed linking 26 of 32 (81.3%) patients and therefore implicating the ward as the location of transmission. Notably, none had outpatient contact or could be linked by residential LSOA and no more than 2 shared the same GP. This is significant because it indicates that current endemic levels are explained by recent transmission on hospital wards that may be missed by a single hospital-centric view, and not elsewhere as has been implied by other studies [3–5]. A comparison of SNP differences between ST22 isolates from outpatient and community settings and inpatients at the same or at different hospitals provides further evidence of ward-based transmission linked to patient sharing between hospitals. This analysis identified less genetic differentiation between inpatient isolates from different hospitals than from a same hospital.

One explanation for these findings may be that once MRSA patients are identified at a particular hospital, that information is recorded on paper and electronic notes enabling immediate implementation of MRSA care pathways locally (contact precautions, isolation, and decolonization) for subsequent admissions; however, only elective admissions and interhospital transfers take place with communication of MRSA status between hospitals. Individuals with MRSA may remain colonized for months or years [26, 27], and test positive even after initial negative screens following decolonization/suppression therapy [28–30]. Thus, a MRSA patient admitted to a second hospital as an emergency would not implement measures immediately on admission if MRSA was under the detection limit and no previous history was documented on site. The risk of direct (patient-to-patient) or indirect transmission would therefore be greater in the second hospital. Patient sharing between hospitals in the absence of equitable sharing of relevant patient data is the most likely explanation for our findings, consistent with observations made in other studies [6–13, 31].

It is important to consider the practical implications of these findings for infection control practice. One simple measure would be for all hospitals in a healthcare network to share data on patient colonization with MRSA and potentially other antimicrobial-resistant bacteria, a measure that has been implemented in some settings [32, 33]. This would allow a MRSA care pathway to be appropriately implemented during emergency admissions. More complex measures such as introduction of discharge screening and WGS services to specifically track transmission would require more data to identify cost-benefits. Indeed, current national policy is to streamline mandatory universal admission screening back to high-risk units only or patients previously known to be MRSA positive [34]; thus, fewer cases will be identified and this will compromise the effectiveness of all targeted measures—the consequences of which remain to be seen.

Strengths include that the laboratories served the vast majority of the population across a healthcare community, universal admission screening was performed at each hospital, and we combined a population-based approach with fine detail of individual patient-ward stays across a hospital network. Limitations include uncertainty over the appropriateness of an SNP cutoff to define transmission [35] and the use of a single patient isolate, which hinders the accurate reconstruction of transmission networks [36]. However, our study was concerned with documenting possible transmission clusters and population-level genetic differentiation and, at this group level, the impact of within-patient genetic diversity is less important [36]. Inferences on transmission events is limited by the short observation period (4 months), the fact that there was no hospital discharge screening, and that we could only analyze discharged patients who returned to a healthcare facility.

Despite successful hospital infection prevention and control programs, our study identified continued ward-based MRSA transmission related to patient sharing between hospitals that underpins a significant proportion of current endemic transmission. We provide evidence that this is due to patients being admitted to multiple hospitals without a process for effective communication of colonization status, an issue that might be relevant for many antimicrobial-resistant pathogens. We suggest that a simple intervention of communicating colonization status across hospitals in a network would potentially limit this transmission although, like all targeted measures, this depends on maintaining an effective screening program for identification.

## Supplementary Data

Supplementary materials are available at *Clinical Infectious Diseases* online. Consisting of data provided by the authors to benefit the reader, the posted materials are not copyedited and are the sole responsibility of the authors, so questions or comments should be addressed to the corresponding author.

## Supplementary Material

Supplementary Material

## Notes

***Author contributions.*** J. D. E., O. T. A., J. B., and T. G. C. conceived and designed the study. J. B., A. I., M. G., R. A. S., J. A. O., and H. M. conducted the laboratory analysis, sample preparation, and sequencing of methicillin-resistant *Staphylococcus aureus* isolates. T. G. C. and R. A. S. analyzed the whole-genome sequencing data; M. D. P. and M. D. contributed to the analysis of these data. O. T. A., R. B., N. D., and T. K. collated and assembled the study metadata. O. T. A. conducted the combined epidemiological analysis of single-nucleotide polymorphism-based clusters and metadata. O. T. A., R. A. S., and J. A. O. wrote the first draft of the manuscript and all authors contributed to the final version. J. D. E., O. T. A., R. A. S., and T. G. C. had full access to all the data and take responsibility for data integrity and accuracy of the analysis. J. D. E., O. T. A., R. A. S., and T. G. C. had full access to all the data in the study and had final responsibility for the decision to submit for publication.

***Acknowledgments.*** We thank Amita Patel for coordination of isolate collection at participant sites and microbiological confirmation of MRSA at the Centre of Clinical Infection and Diagnostics Research.

***Disclaimer.*** The funders had no role in study design, data collection and analysis, decision to publish, or preparation of the manuscript.

***Financial support.*** This research was funded/supported by the National Institute for Health Research (NIHR) Biomedical Research Centre based at Guy’s and St Thomas’ NHS Foundation Trust and King’s College London, program of Infection and Immunity (RJ112/N027) and the NIHR Collaboration for Leadership in Applied Health Research and Care South London at King’s College Hospital NHS Foundation Trust (to J. D. E.). M. D. P. was supported by the Medical Research Council UK (grant numbers MR/K000551/1) T. G. C. also receives funding from the Medical Research Council UK (grant numbers MR/K000551/1, MR/M01360X/1, and MR/N010469/1). J. A. O. acknowledges support from the NIHR Health Protection Research Unit in Healthcare-Associated Infections and Antimicrobial Resistance at Imperial College London in partnership with Public Health England, and the Imperial College Healthcare Trust NIHR Biomedical Research Centre.

***Potential conflicts of interest.*** J. A. O. is a consultant to Gama Healthcare Ltd. All other authors report no potential conflicts. All authors have submitted the ICMJE Form for Disclosure of Potential Conflicts of Interest. Conflicts that the editors consider relevant to the content of the manuscript have been disclosed.
