# Supplementary material for: Frequent Undetected Ward-Based Methicillin-Resistant Staphylococcus aureus Transmission Linked to Patient Sharing Between Hospitals
Source: Clin Infect Dis. 2017 Oct 31;66(6):840–8. doi: 10.1093/cid/cix901 (PMC5850096; doi:10.1093/cid/cix901)

**SUPPLEMENTARY MATERIAL**

**Supplementary Table 1. Summary statistics for pairwise SNPs between and within MRSA MLSTs and hosts and within non-sporadic MLSTs**

|  | **No of Isolates** | | **Min** | **1^st^ Qu.** | **Median** | **Mean** | **3^rd^ Qu.** | **Max** |
| --- | --- | --- | --- | --- | --- | --- | --- | --- |
| Between and within MLSTs | 685 | 0 | | 72 | 9754 | 6729 | 11330 | 16190 |
| Within MLSTs | “ | 0 | | 54 | 66 | 97 | 78 | 15860 |
| Between and within MLSTs within same host | 143 | 0 | | 1 | 3 | 1821 | 67 | 16120 |
| Within same host and MLSTs | “ | 0 | | 1 | 2 | 15 | 5 | 278 |
| ST22 | 408 | 0 | | 54 | 66 | 75 | 77 | 348 |
| ST22 (Catchment boroughs) ^¥^ | 230 | 0 | | 42 | 62 | 68 | 74 | 343 |
| ST36 | 68 | 0 | | 45 | 84 | 74 | 95 | 132 |
| ST8 | 38 | 0 | | 125 | 284 | 233 | 324 | 433 |
| ST5 | 25 | 0 | | 220 | 249 | 276 | 301 | 489 |
| ST1 | 24 | 0 | | 71 | 156 | 158 | 245 | 285 |
| ST88 | 16 | 2 | | 79 | 158 | 144 | 216 | 237 |

^¥^ ST22 isolates from usual residents within catchment boroughs

**Supplementary Table 2. Clusters of genetically related MRSA isolates identified across settings in the cohort when allowing for different SNP cut-offs.**

|  | **SNP Differences** | | | | |
| --- | --- | --- | --- | --- | --- |
|  | 0 | ≤ 5 | ≤ 10 | ≤ 15 | ≤ 20 |
|  |  |  |  |  |  |
| **No. of Clusters** | 61 | 111 | 115 | 109 | 104 |
| **Clusters with > 1 unique patient** | 50 | 87 | 90 | 87 | 85 |
| **Size ^a^** |  |  |  |  |  |
| **2** | 46 | 63 | 55 | 51 | 49 |
| **3** | 3 | 18 | 21 | 19 | 16 |
| **4** |  | 5 | 9 | 5 | 7 |
| **5** |  |  | 4 | 4 | 3 |
| **6** | 1 |  |  | 3 |  |
| **7** |  |  |  | 3 | 2 |
| **8** |  |  |  |  | 4 |
| **10** |  |  |  | 1 | 2 |
| **15** |  | 1 |  |  | 1 |
| **>30** |  |  | 1 | 1 | 1 |
| **Maximum size** | 6 | 15 | 32 | 36 | 37 |
|  |  |  |  |  |  |

A ≤10SNP cut-off identified the largest number of patient clusters (n= 90) and was taken forward for further analysis. **^a^** Cluster size corresponds to the number of unique patients in a cluster.

**Supplementary Figure 1. Distributions of pairwise SNPs between and within MRSA MLSTs and hosts and within ST22s**

**
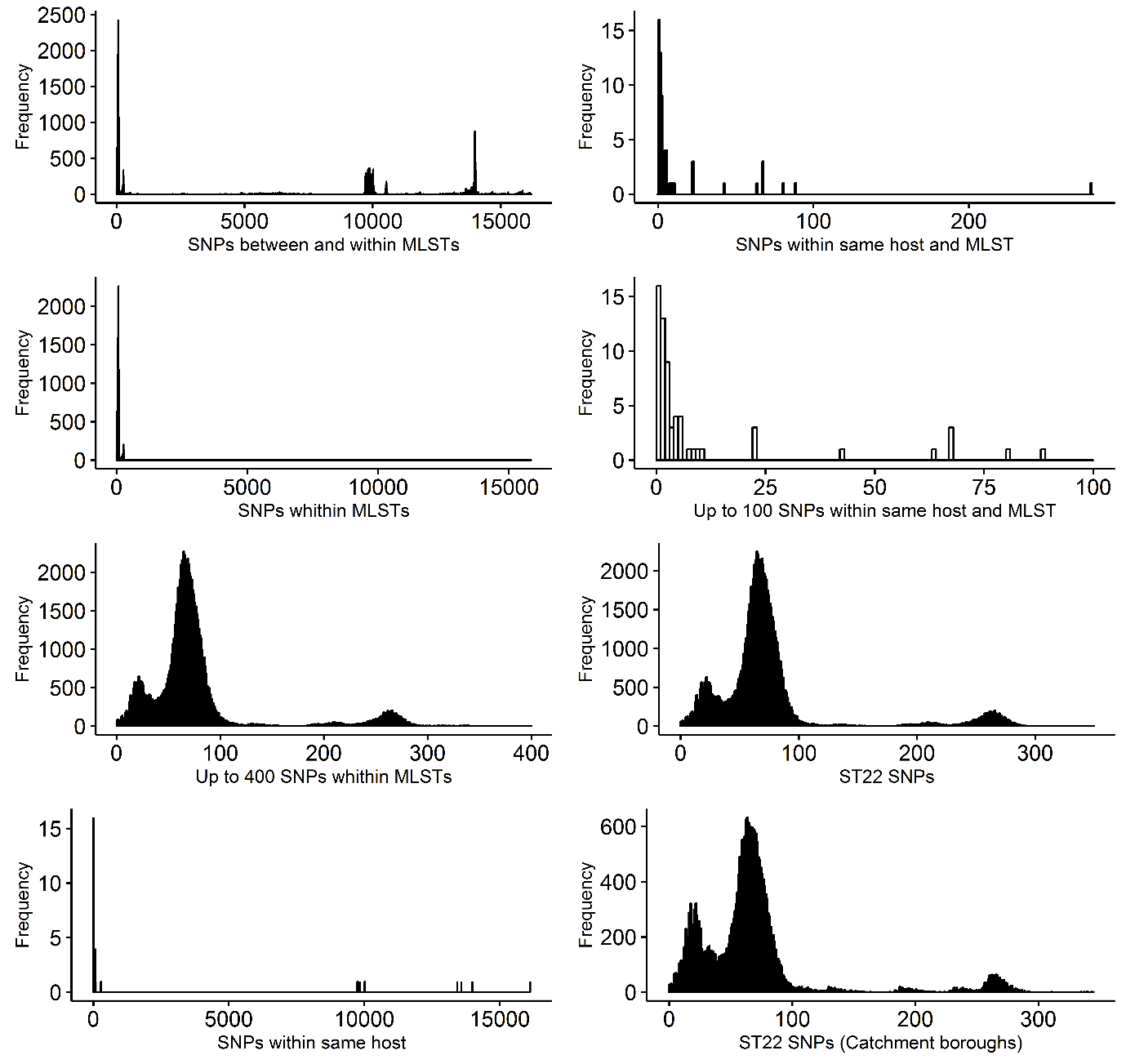
**

**Supplementary Figure 2. SNP-based phylogeny of MRSA isolates.** The SNP population phylogeny for all 685 isolates was constructed using 68,997 genome-wide core SNPs. There are three main clades: **(I)** ST22 (n=408); ST737 (n=4); novel STs that are single SNP variants from ST22 (n=14); (total n=426; 62.2%); **(II)** ST36 (n=68); ST30 (n=9); ST59 (n=8); ST45 (n=3); ST121 (n=3); other (n=2); novel STs that are SNP variants from ST45 (n=3) and ST59 (n=1); (total n=97, 14·2%); **(III)** ST8 (n=38); ST5 (n=25); ST1 (n=24); ST88 (n=16); ST1207 (n=13); ST241 (n=10); ST97 (n=7); ST72 (n=4); ST239 (n=3); ST149 (n=3); ST6 (n=3); other (n=12); novel STs that are SNP variants from ST8 (n=2), ST5 (n=1) and ST361 (n=1); (total n=162, 23·6%). ‘Other’ includes all recognised STs identified from less than 3 isolates. Pie charts show the proportion of isolates in each clade obtained from hospitals A (n=288, orange), B (n=284, blue) or C (n=113, green), and inpatient (n=352), outpatient (n=243) or community (n=90) compartments across the three hospitals. In particular, they show what isolates were obtained from each hospital compartment (hospital A [inpatient [n=171]; outpatient [n=87]; community [n=30]]; hospital B [inpatient [n=151]; outpatient [n=104]; community [n=29]]; hospital C [inpatient [n=30]; outpatient [n=52]; community [n=31]]).


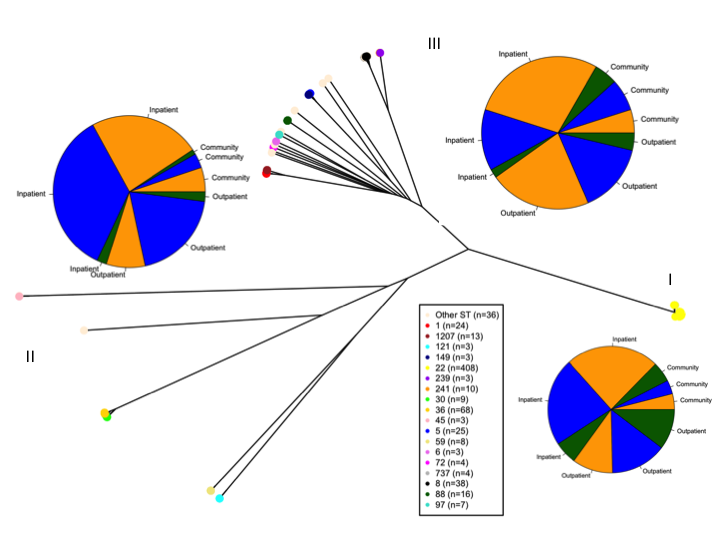

Supplement: Supplementary Material [file cix901_suppl_supplementary_material.docx]
